# What is a reasonable framework for new non-validated treatments?

**DOI:** 10.1007/s11017-020-09537-6

**Published:** 2021-02-14

**Authors:** Gert Helgesson

**Affiliations:** Stockholm Centre for Healthcare Ethics, Karolinska Institutet, Stockholm, Sweden

What to do when established health care treatments do not provide adequate help to patients in great need is a pressing issue for clinical practitioners. Use of non-validated options when standard treatments have been exhausted may be justified in individual cases and has over time led to much progress in medicine [1, 2]. However, such use should take place within some procedural framework protecting patient interests and avoiding decisions primarily driven by the self-interest of the treating physician, clinic, hospital, or commercial agent [2–5].

This need for caution is a leitmotif in Timothy Daly and colleagues’ paper discussing new non-validated interventions for Alzheimer’s disease (AD) [6]. As they point out, there is still no treatment that can make significant improvement to the health of symptomatic AD patients. This also means that there is a great public interest in identifying novel, effective treatments. The authors’ perhaps main concern in their paper is the publication of what they see as grave overstatements by researchers in the field,^1^ and the accompanying risk of luring individual patients and clinicians, and even health care organizations, into believing that reliable options already exist when this is not the case. In drawing attention to these points, they raise important epistemological and ethical issues pertaining to adequate procedures and communication of new non-validated treatment. In what follows, I will deal with some of these issues, with a focus on adequate procedures for use of new non-validated treatment in health care.

## Is the declaration of Helsinki too permissive?

The ethical starting point for the clinic is that established, validated treatments should be used when relevant to cure disease, slow disease development, improve or protect capabilities affected by disease, or alleviate suffering. This position can easily be defended from a utilitarian perspective but can also fit well with duty-based and rights-based approaches to ethics [11]. Non-validated treatments, however, should ideally not be used until they have been validated, due to the uncertainty of benefits and risk of harm [12]. This suggests that research is an imperative in the clinical setting [2]. However, when established treatment options are exhausted and no relevant research projects are available, clinicians and patients face a choice between putting a halt to curative efforts and proceeding into the (partly) unknown.

When and how such a step toward novel treatments should be taken is a topic of debate which has been addressed on several occasions in recent years [2, 5, 13–16]. For instance, two Swedish commission documents were published in the aftermath of the Macchiarini scandal, in which trachea surgery with artificial implants was performed in Sweden, with mortal outcome, as a novel, acclaimed last-resort treatment on patients whose need for last-resort treatment was later questioned [17–20].^2^ According to paragraph 37 of the Declaration of Helsinki, the handling of novel treatments should be very much up to the treating doctor and the concerned patient: “In the treatment of an individual patient, where proven interventions … have been ineffective, the physician, after seeking expert advice, with informed consent from the patient or a legally authorised representative, may use an unproven intervention if in the physician’s judgement it offers hope of saving life, re-establishing health or alleviating suffering” [22]. Daly and colleagues find these criteria too permissive given that the proposal lacks a review system [6]. This claim seems to hinge on how the quite unspecific “seeking expert advice” should be understood—is the declaration referring to formal approval or only stipulating the need for some expert consideration, such as discussing with colleagues or reading a couple of papers?

## Ethical review of treatment in the context of research

Similar to the guidelines for AD innovation outlined by Daly et al. at the end of their paper [6], two Swedish commissions have proposed procedures that involve external review [18, 19]. Both commissions argued that use of urgent non-validated treatment in a single case could be acceptable without including the patient in research, but not in repeated cases; those should instead be handled within an ethically approved clinical study [18, 19]. A similar stance has been taken by the International Society for Stem Cell Research [23]. There are scientific and ethical reasons to favor such an approach. If planned as research from the beginning, use of non-validated interventions is more likely to produce the information needed to draw scientific conclusions. An important ethical benefit of including novel treatments under the rubric of research is that (in many countries) there is already a research-ethical review system in place to conduct external evaluation of their ethical feasibility. With procedures formally disposing non-validated treatments toward a research framework, it would probably also be more difficult for clinical research disguised as mere clinical practice to avoid ethical review [24].

However, there are also potential downsides to applying a strict research approach to novel treatments. First, with regard to rare diseases, there is the risk that many studies will remain unfinished because researchers never manage to gather a sufficient number of cases to allow for meaningful statistical analysis. For such diseases, it could be more pragmatic to require case reports and open storage of data instead of ethically approved clinical trials. At the same time, research-ethical review might be justified even if there never will be a proper study—this seems to be the position recently argued for by Jake Earl [2]. Second, since an essential aspect of research protocols is strictness (every individual in the same study arm, experiment group or control group, should receive the same treatment), adjustments in accordance with the needs of an individual patient are disfavored. If the justification for initiating a research project was to have a final chance to successfully treat a patient, then it would seem unfortunate that the research protocol, rather than the patient’s individual needs, should steer what exact measures are taken. Nonetheless, it is difficult to judge the weight of this particular argument considering that a reasonable objective of introducing new treatments at the clinic is to find out if they work. Third, if treatment needs are urgent, then it is not always tenable to wait for a response from the ethical review board, so it is questionable whether research-ethical approval should be mandatory in order to distribute a treatment in very pressing cases. Even if there is time to await the verdict of research-ethical review, it is debatable why the board’s decision should be followed in the particular case at hand. Treatment decisions should arguably be made by health care providers and not by research-ethical review boards. The response from the review board should be decisive regarding planned research but is not reasonably treated as the final word when it comes to decisions about individual patients [24]. To summarize, even though the idea of initiating research when more patient cases are to be expected is laudable, as in the case of Alzheimer’s disease, such an approach must not be allowed to hinder treatment or the spreading of information where patients in need of a specific novel treatment are rare.

## Procedure for new non-validated treatments at the hospital level

Daly and colleagues call for external review of new non-validated treatments, since they believe that it is not right to leave decisions about novel interventions entirely up to individual doctors and consenting patients [6], a view supported by others [1, 2, 5, 18, 19]. But if it is not right that research-ethical review boards have the final say on whether or not to initiate novel treatment in individual cases either, then who should? The head of the clinic seems to be a natural choice, as a way of moving decisions about approval away from treating doctors; the introduction of novel treatments at the clinic is, after all, a concern for the clinic and not just for individual clinicians. Discussion among senior physicians at the clinic is needed in any case when something out of the ordinary is considered—options should be evaluated, including weighing the pros and cons of the suggested novel treatment for the patient in question. One means of assisting reflection on the justification for introducing new non-validated treatments could be to appoint a hospital advisory board dedicated to such considerations, preferably including legal and ethical experts in addition to one or more senior physicians.^3^

The Macchiarini scandal, mentioned above, took place within Swedish public health care. Should the procedure for handling new non-validated treatments be any different in a private for-profit hospital? I think not. There might be an implicit presumption in asking this question that in for-profit contexts there is an even greater risk that considerations other than the best interest of the patient will influence the decision-making process. It is beyond the scope of this commentary to evaluate such risk, but if the presumption is correct, then the problem probably permeates choices at all levels of health care provision and not specifically new non-validated practice, which in turn suggests that it needs to be politically dealt with at a much more basic level. However, it is conceivable that a health care system that emphasizes consumer choice more strongly than medical need might be inclined to give greater weight to patient wishes than to medical expertise in pressing situations.^4^ Since wishes sometimes rest on false hope and misrepresentations about the likelihood of success, it remains crucial that doctors listen, inform, and ensure realistic expectations of outcomes.

## Reasonable grounds for thinking a novel treatment will work

That a treatment is not validated means that there is no firm evidence regarding its effects. However, this is not to say that nothing speaks either in favor of or against its use. If there were no precedents of any kind, practical or theoretical, then the treatment would be a total blind shot—introducing novel treatments could never be recommended on that basis. It is reasonable to require some support for novel treatments; the more research documentation, the better. One needs to know not only about expected treatment effects, but also about positive and negative side effects. Patient involvement is necessary when it comes to evaluating what steps could be worth taking.

Sometimes when novel treatments are considered, only a few clinical studies have been published—or, worse, only a few case studies—and there is limited clinical experience. Then information from existing data in combination with good theoretical reasons, preferably relating to a specific mechanism, is needed. If there is no empirical support concerning the specific treatment and medical context at hand, then this dearth offers strong reason not to use the novel treatment; but at times there may nevertheless be reason to believe it would work, either because it has worked in other contexts that involve the same kind of mechanism or because there is a good theoretical basis for thinking such an intervention could be effective. This could be acceptable grounds for approving new non-validated treatments in some cases—where the patient is willing to take the chance and the treating doctor’s and concerned hospital’s well-considered judgment is that the treatment might work and provide the patient some improvement. When one talks about new non-validated treatments, uncertainty is unavoidably at the heart of the matter. Ultimately, once all the available evidence has been considered, reflective, reciprocal, shared professional judgment is all the medical basis for a decision there is.

Let me provide one example of what a decision situation might be like: In Stockholm a committee evaluating use of non-validated treatments at one of the larger hospitals approved treatment in a case where a patient was on his way to starving to death because his intestines were unable to take up nourishment properly. No identical precedents were found in the literature, but there were a few documented cases involving patients with very short intestines (which also reduces the ability to take up nourishment), where a certain medication was able to increase uptake. There were no observed negative side effects. The suggestion from the clinic was to provide the present patient with the medication and observe the effects closely. The only perceived alternative option at that stage was palliative care.

As argued by Dominic Wilkinson and Julian Savulescu, the expected utility of a treatment does not have to be positive in the individual case at hand if the patient is a competent consenting adult [15]. Perhaps one should still insist that the procedural framework applied for new non-validated treatments should be such that their utility can reasonably be expected to be positive. This condition could be met if the procedural framework leaves room for exploration and innovation in the clinical context, with certain restraints, which means that new effective treatments can be the useful output in the long run.

## Conclusions

There are conflicting interests relating to new non-validated treatments. On the one hand, health care providers can do much good by identifying novel working treatments when the established ones fail. On the other hand, patients must not become guinea pigs for improvising clinicians or status-searching clinics. A procedural framework for the handling of new non-validated treatments needs to be in place. As a starting point, support for novel treatments should primarily rest on systematic research in the form of clinical trials. However, getting an approved clinical trial in place is not always feasible in urgent last-resort cases or when a disease is very rare. In such cases, decisions about whether or not to proceed with a novel treatment, if the patient approves, are clinical, not research-ethical; they should be made at the clinic after due consideration of existing empirical and theoretical knowledge.

## Footnotes

1 Specifically, they look at overstatements regarding what they term metabolic enhancement protocols as advanced by Dale Bredesen and colleagues [7–10]. I leave this particular issue aside here, but welcome AD researchers to join the critical discussion.

2 Previous investigations never led to prosecution, but recently a legal case was opened based on the understanding that Paolo Macchiarini’s trachea surgery was neither legal research nor acceptable treatment [21].

3 Different solutions may be feasible under different legislation. But where legal responsibility rests with the clinic and/or the treating doctor, final decisions cannot be made by other bodies. Decisions not to try a novel treatment should primarily lie with the treating doctor, ideally after shared decision-making with the patient.

4 To avoid possible misunderstandings: there is no necessary connection between for-profit health care and a consumer-choice approach to health care delivery. There are for-profit hospitals in Sweden, but all health care provision in the country is legally obliged to focus on patient needs, rather than patient demands.
